# Genomic portfolio of Merkel cell carcinoma as determined by comprehensive genomic profiling: implications for targeted therapeutics

**DOI:** 10.18632/oncotarget.8032

**Published:** 2016-03-10

**Authors:** Philip R. Cohen, Brett N. Tomson, Sheryl K. Elkin, Erica Marchlik, Jennifer L. Carter, Razelle Kurzrock

**Affiliations:** ^1^ Department of Dermatology, University of California San Diego, San Diego, CA, USA; ^2^ N-of-One, Inc., Lexington, MA, USA; ^3^ Center for Personalized Cancer Therapy and Division of Hematology and Oncology, Department of Medicine, University of California San Diego Moores Cancer Center, San Diego, CA, USA

**Keywords:** merkel cell carcinoma, next-generation sequencing, targeted therapy, personalized therapy, genomic landscape

## Abstract

Merkel cell carcinoma is an ultra-rare cutaneous neuroendocrine cancer for which approved treatment options are lacking. To better understand potential actionability, the genomic landscape of Merkel cell cancers was assessed. The molecular aberrations in 17 patients with Merkel cell carcinoma were, on physician request, tested in a Clinical Laboratory Improvement Amendments (CLIA) laboratory (Foundation Medicine, Cambridge, MA) using next-generation sequencing (182 or 236 genes) and analyzed by N-of-One, Inc. (Lexington, MA). There were 30 genes harboring aberrations and 60 distinct molecular alterations identified in this patient population. The most common abnormalities involved the *TP53* gene (12/17 [71% of patients]) and the cell cycle pathway (*CDKN2A/B*, *CDKN2C* or *RB1*) (12/17 [71%]). Abnormalities also were observed in the PI3K/AKT/mTOR pathway (*AKT2, FBXW7, NF1, PIK3CA, PIK3R1, PTEN* or *RICTOR*) (9/17 [53%]) and DNA repair genes (*ATM, BAP1, BRCA1/2, CHEK2, FANCA* or *MLH1*) (5/17 [29%]). Possible cognate targeted therapies, including FDA-approved drugs, could be identified in most of the patients (16/17 [94%]). In summary, Merkel cell carcinomas were characterized by multiple distinct aberrations that were unique in the majority of analyzed cases. Most patients had theoretically actionable alterations. These results provide a framework for investigating tailored combinations of matched therapies in Merkel cell carcinoma patients.

## INTRODUCTION

Merkel cell carcinoma is an extremely uncommon, biologically aggressive, cutaneous neuroendocrine cancer [1–4]. It typically presents on sun-exposed skin of elderly men as a rapidly enlarging asymptomatic flesh-colored or blue-red nodule. Local, regional, and distant recurrences are associated with a poor prognostic outcome.

Management for localized disease is surgery: a wide local excision and a sentinel lymph node biopsy. A complete lymph node dissection may follow for patients with a positive sentinel lymph node for cancer. In addition, adjuvant radiation therapy is usually given not only to patients with positive sentinel lymph nodes, but also to patients with Merkel cell carcinoma of the head and neck [1–4].

For patients with metastatic disease, chemotherapy is used. Unfortunately, after two to three cycles of treatment, resistance frequently develops. In addition to radiation therapy [5, 6], immunotherapy (such as systemic pembrolizumab [MK-3475] a humanized anti-PD1 antibody [7]) and targeted molecular therapy are investigational approaches that have been used for metastatic Merkel cell carcinoma [8–11].

Due to the rarity of the disease, data regarding response to therapy are often derived from case reports and retrospective series, rather than prospectively performed clinical trials. Thus, it has been challenging to define the role of chemotherapy in management of advanced Merkel cell carcinoma. Systemic chemotherapies currently used include platinum with or without etoposide, as well as cyclophosphamide, doxorubicin and vincristine [3–5]. Modest responses can be achieved with these cytotoxic agents (median progression-free survival of 3 months). Indeed, there are no drugs approved by the Food and Drug Administration (FDA) specifically for Merkel cell carcinoma.

Importantly, in Merkel cell carcinomas, several molecular abnormalities have been reported [12–30]. These include overexpression of Hedgehog (Hh) signal pathway proteins, telomerase activation (*TERT*), tumor suppressor anomalies (*TP53*, *RB1* and *SUFU*), and tyrosine kinase signaling activation (*AKT*, *KIT*, *PDGFRA*, *PIK3CA* and *PTEN*). In addition, chromosomal abnormalities [29] and microRNA alterations [30] have been demonstrated in Merkel cell carcinomas.

Clinical trials using a variety of targeted tyrosine kinase inhibitors, either as monotherapy or in combination with chemotherapy or one or more additional tyrosine kinase inhibitors, have been initiated for Merkel cell carcinoma. Although a complete response with imatinib (targeting KIT and PDFGR) has been described [31], a low response rate to the agent was observed in a clinical trial [32]. Similarly, a complete response to pazopanib has been observed in Merkel cell carcinoma resistant to chemotherapy [33]; currently, a phase II trail (NCT01841736) is open to evaluate pazopanib in patients with neuroendocrine tumors including Merkel cell carcinoma. However, for several of the current trials, in which these therapies are being given to unselected patients rather than matched to individuals whose tumors harbored cognate aberrations, the results have yet to be reported. Indeed, we are unaware of any trials in which Merkel cell carcinoma patients Merkel cell carcinoma were selected for the presence of specific aberrations and were treated with appropriated targeting agents.

Given that additional effective treatment strategies are needed, the genomic profiles of Merkel cell carcinomas, as determined by comprehensive genomic profiling (targeted next-generation sequencing (NGS)), were examined and the data analyzed in the context of potential actionability.

## RESULTS

### Genetic aberrations in Merkel cell carcinomas (Tables 1 and 2, Figure 1)

**Table 1:** enomic portfolio in each of 17 patients with Merkel cell carcinoma [a]

C	Aberrations	No. of genealterations per patient [a]	Cell cycle pathway	DNA repair gene	PI3K/AKT/mTORpathway	Potentially actionable
1	NF1 L937*	3			X	Yes
	RB1 Q685*		X			No
	TP53 H179Y					Yes
2	RICTOR amplification	1			X	Yes
3	CDKN2C loss	2	X			Not clear
	PIK3R1 Q221*				X	Yes
4	BAP1 G422fs*8	5		X		Yes
	BRCA2 K3326*			X		Yes
	PDGFRB L986F [b]					Yes
	RB1 Q257*		X			No
	TP53 C275W					Yes
5	ARID1A loss	1				No
6	MYC amplification	4				No
	NTRK3 K461R [b]					Yes
	RB1 Q93*		X			No
	TP53 K120*					Yes
7	AKT2 amplification	3			X	Yes
	RB1 Q93*		X			No
	TP53 Q331*					Yes
8	CDKN2A/B loss	2	X			Yes
	EGFR E282K [b]					Yes
9	BAP1 Q729*	5		X		Yes
	FANCA T1161M [b]			X		Yes
	MLH1 E694*			X		Yes
	RB1 splice site 1499 − 2A > G RB1 splice site 2489 + 1G > A TP53 R248W		X X			No No Yes
10	FBXW7 Q95*	5			X	Yes
	NOTCH1 splice site 4586 + 1G > A [c]		X			Yes
	RB1 splice site 1422 − 1G > A					No
	SMARCA4 R1192C					No
	TP53 R280K					Yes
11	KMT2D truncation, exon 4	4				Not clear
	NOTCH1 splice site 5168 − 1G > A [c]					Yes
	RB1 A392fs*5		X			No
	TP53 R175H					Yes
12	ATM R2993*	4		X		Yes
	NOTCH1 E256* [c]					Yes
	RB1 S249*		X			No
	TP53 R282W					Yes
13	BRCA1 Q1756*	4		X		Yes
	PIK3CA E542K				X	Yes
	PTEN splice site 635 − 1G > A				X	Yes
	TP53 E339K					Yes
	TP53 G187S					Yes
	TP53 R202fs*45					Yes
14	ALK F1174CRET E511K	2				YesYes
15	CHEK2 R346G [b]	3		X		Yes
	PIK3CA R88Q				X	Yes
	TP53 P177L					Yes
16	PIK3CA G1049R	4			X	Yes
	PTCH1 P369L [b]					Yes
	RB1 M386fs*1		X			No
	TP53 R224H					Yes
	TP53 Y220*					Yes
17	APC W2612*	5				Yes
	EPHAS R417Q [b]					Not clear
	NF1 splice site 5609 + 1G > A				X	Yes
	RB1 W99*		X			No
	TP53 P151S					Yes
	TP53 R248W					Yes

Abbreviations: C, case; No., number.

[a] 4 cases had more than one molecular aberration in the same gene: case 9 [*RB1* = 2], case 13 [*TP53* = 3], case 16[*TP53* = 2], and case 17 [*TP53* = 2].

[b] Aberration is of uncertain clinical significance and relevance of therapeutic strategies is unknown.

[c] Aberration is an inactivating alteration and therapeutic strategies are not expected to be relevant.

**Table 2:** Summary of genomic alterations in patients with Merkel cell carcinoma

Aberration	Number of patients	Percent of patients	Potentially actionable [a]
TP53	12	71	Yes
RB1	10	59	No
NOTCH1	3	18	No [b]
PIK3CA	3	18	Yes
BAP1	2	12	Yes
BRCA1/2	2	12	Yes
NF1	2	12	Yes
AKT2	1	6	Yes
ALK	1	6	Yes
APC	1	6	Yes
ARIDIA	1	6	No
ATM	1	6	Yes
CDKN2A/B	1	6	Yes
CDKN2C	1	6	Not clear [c]
CHEK2	1	6	Yes
EGFR	1	6	Yes
EPHAS	1	6	Not clear [c]
FANCA	1	6	Yes
FBXW7	1	6	Yes
KMT2D	1	6	Not clear [c]
MLH1	1	6	Yes
MYC	1	6	No
NTRK3	1	6	Yes
PDGFRB	1	6	Yes
PIK3R1	1	6	Yes
PTCH1	1	6	Yes
PTEN	1	6	Yes
RET	1	6	Yes
RICTOR	1	6	Yes
SMARCA4	1	6	No

[a] Potentially actionable indicates some evidence in the literature that there are drugs that impact the target. This evidence may derive from clinical observations in other tumors or from preclinical evidence.

[b] Activating *NOTCH* mutations are potentially actionable but the ones in this series were inactivating.

[c] Not clear indicates mixed or inconclusive literature evidence for the potential of available drugs to impact the altered gene product.

**Figure 1:**
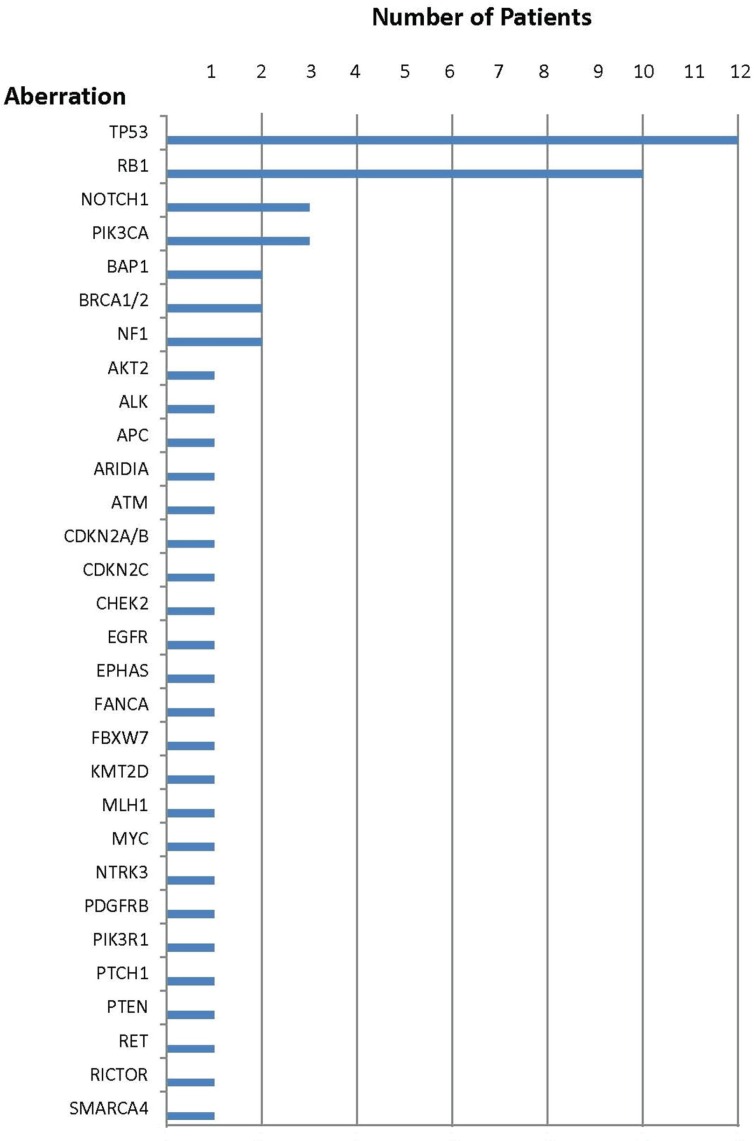
Number of patients with each aberration

Specific genomic abnormalities were observed in all 17 Merkel cell carcinomas and ranged from one to five alterations per tumor; the median was four. Only two patients (cases 2 and 5) had one aberration and only three patients (cases 1, 7 and 15) had two aberrations. Indeed, more than half of the patients (9/17 [53%]) had four or more genetic anomalies.

The most common anomaly among all Merkel cell carcinomas was in the *TP53* gene (12/17 patients [71%]). Abnormalities in the cell cycle pathway (*CDKN2A/B, CDKN2C* or *RB1*) were also observed in 71% of cases [12/17]. Aberrations in the PI3K/AKT/mTOR pathway (*AKT2, FBXW7, NF1, PIK3CA, PIK3R1, PTEN* or *RICTOR*) were the third most common set of aberrations (9/17 [53%]). Anomalies in DNA repair genes (*ATM, BAP1, BRCA1/2, CHEK2, FANCA* or *MLH1*) were seen in 29% (5/17) of patients. Aberrations in either *ALK* and *RET* (case 14) or *ARIDIA* (case 5) were each only noted in 6% (1/17) of patients.

Concurrent anomalies in both the cell cycle and PI3K/AKT/mTOR pathways were noted in 35% (6/17) of patients (cases 1, 3, 7, 10, 16 and 17). Abnormalities of both the cell cycle pathway and DNA repair genes occurred in 18% (3/17) of patients (cases 4, 9 and 12) and aberrations in PI3K/AKT/mTOR pathway and DNA repair genes were discerned in 12% (2/17) of patients (cases 13 and 15).

### Number of genomic aberrations and the distinctness of the profiles (Tables 1 and 2)

There were 30 distinct genes involved with 60 distinct molecular alterations. Genomic twins refer to two or more patients that have alterations in the identical genes. Molecular twins refer to two or more patients that have alterations in the same genes and the specific alterations within the gene are also identical. There were no genomic or molecular twins in this study. Therefore, our analysis showed that each of the 17 Merkel cell carcinomas were distinct at the genomic and at the molecular level.

### *TP53* suppressor gene aberrations (Tables 1 and 2, Figure 1)

Genomic abnormalities in *TP53* were found in 71% (12/17) of patients. However, amongst the 16 molecular aberrations, 15 were distinct; two patients (cases 9 and 17) had the same molecular abnormality: R248W. One tumor (case 13) harbored three distinct molecular *TP53* abnormalities and two tumors (cases 16 and 17) harbored two distinct molecular aberrations.

### Cyclin pathway aberrations (Tables 1 and 2, Figure 1)

Aberrations in cell cycle genes were observed in 71% (12/17) of patients. The most common aberration was in the *RB1* tumor suppressor gene; *RB1* was mutated in 10 patients. In one patient (case 9), there were two molecular aberrations in *RB1*. Genomic alterations in either *CDKN2A/B* (case 8) or *CDKN2C* (case 3) were each observed in one patient.

### PI3K/AKT/mTOR pathway aberrations (Tables 1 and 2, Figure 1)

Genomic abnormalities in the PI3K/AKT/mTOR pathway were noted in 53% (9/17) of patients. There were 10 molecular abnormalities in these nine patients; one patient (case 13) had an aberration in both *PIK3R1* and *PTEN*. The most common genomic aberration was in *PIK3CA* [found in three patients (cases 13, 15 and 16)]; two patients had a genomic aberration of the *NF1* gene.

### DNA repair gene aberrations (Tables 1 and 2, Figure 1)

DNA repair gene abnormalities were observed in 29% (5/17) of patients. They included eight molecular abnormalities. Two patients had genomic aberration of *BAP1* (cases 4 and 9); two patients had *BRCA1/2* alterations (case 13 had a *BRCA1* abnormality and case 4 had a *BRCA2* abnormality). In two of the five patients, there were abnormalities in multiple DNA repair genes; either *BAP1* and *BRCA2* (case 4) or *BAP1*, *FANCA*, and *MLH1* (case 9). Genomic alterations in either *ATM* (case 12) or *CHEK2* (case 15) were each observed in one patient.

### Actionable aberrations (Tables 1, 2 and 3)

**Table 3:** Potential therapies for genomic aberrations in each of 17 patients with Merkel cell carcinoma [34–72] [a]

C	Aberrations	Examples of potential cognate targeted therapies
1	NF1 L937*	NF1 may be targeted with the mTOR inhibitor everolimus [34, 35] and /or the MEK inhibitor trametinib [36]
	RB1 Q685*TP53 H179Y	Longer progression free survival with bevacizumab in patient with TP53 mutations [37, 38]
2	RICTOR amplification	RICTOR amplification is targetable by investigational mTORC1/mTORC2 inhibiotrs (such as AZD8055 and MLN0128) [39–41]
3	CDKN2C lossPIK3R1 Q221*	PIK3R1 mutation targeted with mTOR inhibitor everolimus [42, 43]
4	BAP1 G422fs*8	BAP1 targeted with PARP inhibitor olaparib [44–47]
	BRCA2 K3326*	BRCA2 targeted with PARP inhibitor olaparib [44, 45, 48, 49]
	PDGFRB L986F [b]	PDGFRB targeted by dovitinib [50] and sorafenib [51]
	RB1 Q257*TP53 C275W	Longer progression free survival with bevacizumab in patient with TP53 mutations [37, 38]
5	ARID1A loss	
6	MYC amplificationNTRK3 K461R [b]	NTRK3 inhibitors in development; also targeted by crizotinib [52, 53]
	RB1 Q93*TP53 K120*	Longer progression free survival with bevacizumab in patient with TP53 mutations [37, 38]
7	AKT2 amplification	AKT2 may be targeted with AKT or mTOR inhibitors [54] or MEK inhibitors [55]
	RB1 Q93*TP53 Q331*	Longer progression free survival with bevacizumab in patient with TP53 mutations [37, 38]
8	CDKN2A/B loss	CDKN2A/B loss leads to activation of the CDK4/6 pathway which can be targeted with CDK4/6 inhibitor palbociclib [56]
	EGFR E282K [b]	EGFR targeted with erlotinib or cetuximab [57]
9	BAP1 Q729*	BAP1 may theoretically be targeted by olaparib and platinums [44–47]
	FANCA T1161M [b]	FANCA may theoretically be targeted by PARP inhibitors and platinums [44–47, 58, 59]
	MLH1 E694*	MLH1 mutations may be targeted by PARP inhibitors and Top1 inhibitor (irinotecan) [60] or antiPD1 agents [61]
	RB1 splice site 1499 – 2A > GRB1 splice site 2489 + 1G > ATP53 R248W	Longer progression free survival with bevacizumab in patient with TP53 mutations [37, 38]
10	FBXW7 Q95*	FBXW7 may be targeted by mTOR inhibitors [62, 63]
	NOTCH1 splice site 4586 + 1G > A [c]	NOTCH1 is potentially targetable with gamma-secretase inhibitor [64, 65]; this alteration is unlikely to be activating
	RB1 splice site 1422–1G > ASMARCA4 R1192C^1^TP53 R280K	Longer progression free survival with bevacizumab in patient with TP53 mutations [37, 38]
11	KMT2D truncation, exon 4NOTCH1 splice site 5168–1G > A [c]	NOTCH1 is potentially targetable with gamma-secretase inhibitor [64, 65]; this alteration is unlikely to be activating
	RB1 A392fs*5TP53 R175H	Longer progression free survival with bevacizumab in patient with TP53 mutations [37, 38]
12	ATM R2993*NOTCH1 E256* [c]	ATM mutation targeted with olaparib [66] NOTCH1 is potentially targetable with gamma-secretase inhibitor [64,65]; this alteration is unlikely to be activating
	RB1 S249*TP53 R282W	Longer progression free survival with bevacizumab in patient with TP53 mutations [37, 38]
13	BRCA1 Q1756*PIK3CA E542K	BRCA1 targeted with PARP inhibitor olaparib [48] PIK3CA mutations may be targeted with the mTOR inhibitor everolimus [42, 43]
	PTEN splice site 635–1G > A	PTEN mutations may be targeted with the mTOR inhibitor everolimus [43]
	TP53 E339KTP53 G187STP53 R202fs*45	Longer progression free survival with bevacizumab in patient with TP53 mutations [37, 38]
14	ALK F1174C	ALK targeted with crizotinib [67]
	RET E511K	RET targeted with cabozantinib [68]
15	CHEK2 R346G [b]PIK3CA R88Q	CHEK2 may be targeted by olaparib and platinums [44–47, 69] PIK3CA mutations may be targeted with the mTOR inhibitor everolimus [42, 43]
	TP53 P177L	Longer progression free survival with bevacizumab in patient with TP53 mutations [37, 38]
16	PIK3CA G1049R	PIK3CA mutations may be targeted with the mTOR inhibitor everolimus [42, 43]
	PTCH1 P369L [b]	PTCH1 mutation targetable with vismodegib [70]
	RB1 M386fs*1TP53 R224HTP53 Y220*	Longer progression free survival with bevacizumab in patient with TP53 mutations [37, 38]
17	APC W2612*	APC may be targeted with sulindac [71, 72]
	EPHA5 R417Q [b]	
	NF1 splice site 5609 + 1G > A	NF1 may be targeted with the mTOR inhibitor everolimus [34, 35] and/or the MEK inhibitor trametinib [36]
	RB1 W99*TP53 P151STP53 R248W	Longer progression free survival with bevacizumab in patient with TP53 mutations [37, 38]

Abbreviations: C, case.

[a] Many of these therapies have not been validated as effective in patients.

[b] Aberration is of uncertain clinical significance and relevance of therapeutic strategies is unknown.

[c] Aberration is an inactivating alteration and therapeutic strategies are not expected to be relevant.

Of the 30 distinct genomic aberrations, 73% (22/30) were theoretically targetable by either an off-label use of an FDA-approved drug (21/30) or an experimental drug in a clinical trial where an off-label use did not exist (1/30).

The vast majority of patients (94%, 16/17) had at least one aberration that was potentially targetable. There were between zero (case 5) and four (cases 4, 9 and 13) actionable genes affected per patient (median, two genes per patient). Potential therapies for the genomic aberrations in each of the 17 patients with Merkel cell carcinoma are summarized in Table 3 [34–72].

## DISCUSSION

Merkel cell carcinoma is an ultra-rare neuroendocrine cancer of the skin that most commonly presents in elderly Caucasian men [5, 73]. The pathogenesis is related not only to ultraviolet light exposure, but also to immunosuppression [5, 73]. In addition, the presence of Merkel cell polyomavirus (MCPyV) has been demonstrated in about 45% [73] to 80% [74] of the cases. Gene mutations may have a role in the etiology of Merkel cell carcinoma, particularly in patients whose tumors are Merkel cell polymavirus-negative [75]. A recent study of nine virus-negative patients showed high mutational burden (as compared to that in virus-positive patients), and alterations in *TP53, RB1, PIK3CA, HRAS, PRUNE2* and *NOTCH* (integrative sequencing that included data from whole-exome sequencing and whole-transcriptome sequencing) [13]. Another similar study (*N* = 619 genes analyzed; 21 virus-negative and 13 virus-positive patients) confirmed high mutation burden and a UV-induced DNA damage signature for virus-negative patients. All viral-negative tumors harbored mutations in *RB1, TP53,* and a high frequency of mutations in *NOTCH1* and *FAT1*. Additional mutated or amplified cancer genes of potential clinical importance included those in the PI3K or MAPK pathway [14]. Of interest, a subset of virus-negative patients showed high PDL1, suggesting that they might respond to antiPD1 checkpoint inhibitors [15].

The prognosis for patients with Merkel cell carcinoma is poor; more than 33% of patients die from their disease and 50% of patients with advanced tumors live less than 9 months following diagnosis [76]. Of interest in this regard is that exome sequencing of Merkel cell revealed that *TP53* was more common in the virus-negative group and predicted a poor survival (5-year survival in *TP53* mutant versus wild-type stage I and II disease was 20% vs. 92%, respectively; *P* = 0.0036) [16]. In general, Merkel cell carcinoma has shown low response rates to chemotherapy [4-6] and to molecularly targeted therapies that are administered without molecular matching [32]. Thus, therapeutic options for Merkel cell carcinoma are limited. In addition, we are not aware of any reports that describe the response in Merkel cell carcinomas when genetic aberrations and therapies were matched. We therefore investigated the genomic landscape of Merkel cell carcinomas by comprehensive genomic profiling and analyzed potential pharmacologic tractability.

The most common genetic aberration among 17 patients with Merkel cell carcinoma was *TP53* mutation (12/17 [71%]) (Tables 1 and 2, Figure 1). Our current study observed a markedly higher incidence of *TP53* mutations than that noted in previous reports that demonstrated *TP53* mutations ranging from 0% to 37% [16, 19, 20, 77, 78]. The *TP53* gene is large and there are many areas that can be mutated [79]; our study used comprehensive genomic profiling that evaluated all areas of the gene; in contrast, some of the earlier reported results sequenced discrete regions of the *TP53* gene and may not have identified all existing mutations. *TP53* gene anomalies are generally seen in virus-negative Merkel cell cancers [16], but a limitation of our study is that viral status was not available. Finally, each of the reports of Merkel cell genomics have small numbers of patients, perhaps accounting in part for the variability in percent positive for *TP53* mutations.

*TP53* has proven difficult to target. MDM2 inhibitors can theoretically be used in patients with wild-type *TP53*. Recent data suggest that *TP53* mutations result in increased levels of VEGFA, which is the target of bevacizumab [80]. Said et al. showed that bevacizumab-containing regimens were associated with longer progression-free survival when compared to non-bevacizumab-containing regimens in patients with *TP53*-mutated advanced solid tumors (median 11.0 versus 4.0 months (*p* < 0.01) [37]. Wee-1 inhibitors, which are in experimental trials, may also target *TP53* [81] (Table 3 [34–72]).

The cell cycle pathway (*CDKN2A/B, CDKN2C* or *RB1* genes) was also abnormal in 71% of patients (12/17) with Merkel cell carcinomas (Tables 1 and 2, Figure 1). Aberrations in the cyclin D-cyclin-dependent kinase pathway that regulates the cell cycle restriction point is a common feature of human cancer, contributing to tumor proliferation, genomic instability and chromosomal instability [12, 82, 83]. This pathway can be altered through multiple mechanisms including increased signaling through *CDK4* and *CDK6* amplification, overexpression of cyclin D1, and loss of inhibitors including *CDKN2A* and/or *CDKN2B* [84–87]. Regarding therapeutic implications, the cell cycle pathway is possibly targetable with CDK4/6 inhibitors such as palbociclib [56], and further investigation is warranted (Table 3 [34–72]).

Mutation or loss of *RB1*, a tumor suppressor gene, also alters the cell cycle pathway. *RB1* gene alterations in Merkel cell cancers are associated with virus-negative disease [88]. Merkel cell polyomavirus large-T antigen binds RB1 with high affinity, suppressing its anti-neoplastic function [89]. Aberration of *RB1* renders tumors resistant to CDK4/6 inhibitors such as palbociclib [38]. Ten patients in our series had *RB1* mutations (Tables 1 and 2, Figure 1).

Importantly, aberrations in the PI3K/AKT/mTOR pathway (*AKT2, FBXW7, NF1, PIK3CA, PIK3R1*, *PTEN* or *RICTOR*) were also commonly seen in Merkel cell carcinomas (9/17 [53%]) (Tables 1 and 2, Figure 1). *PIK3CA* is a key regulator of cell motility and chemotaxis. Aberrations in *PI3KCA* usually occur in tumors that do not have Merkel cell polyomavirus [24, 25].

The PI3K/AKT/mTOR pathway can be targeted by PI3K/AKT/mTOR inhibitors such as everolimus and temsirolimus, both of which are FDA-approved mTOR inhibitors [42, 43]. Since Merkel cell carcinomas—regardless of whether they are positive or negative for Merkel cell polyomavirus—show activated PI3K/AKT signaling, PI3K and dual PI3K/mTOR inhibitors may be used as potential targeted therapies, though the literature suggests that for many tumors with pathway activation, they are not effective as single agents [24, 25]. With regards to *RICTOR* amplification, recent studies have shown that this aberration may be targetable by investigational mTORC1/mTORC2 inhibitors such as AZD8055 and MLN0128 [39–41] (Table 3 [34–72]).

Several investigators have also previously shown that MAP (mitogen-activated protein) kinase-related genes—such as *KRAS* and *BRAF*—are more frequently aberrant in the presence of mutant *PIK3CA*, as compared with wild-type *PIK3CA* [90]. These genes may confer resistance to PI3K/AKT/mTOR inhibitors. Interestingly, none of our patients had *KRAS* or *BRAF* alterations.

Abnormalities in the DNA repair gene pathway (*ATM, BAP1, BRCA1/2, CHEK2, FANCA* or *MLH1*) were also observed in 29% of patients (5/17) (Tables 1 and 2, Figure 1). Drugs such as platinums, PARP inhibitors, and possibly immunotherapeutic agents can target DNA repair gene abnormalities (Table 3 [34–72]). Some of these abnormalities (such as *BRCA1/2* or *ATM*) can be germline; germline testing was not conducted in the patients included in this analysis.

Interestingly, 16**/**17 patients (94%) had potentially actionable aberrations (Table 1). The number of actionable genes affected per patient ranged between zero (case 5) and four (cases 4, 9 and 13), with a median of two per patient. Indeed, the majority of the genomic alterations were theoretically druggable (Tables 1 and 2). Of the 22 (73%) actionable aberrations, 21 were targetable by an FDA-approved drug (off-label) (representing 70% [21/30] of all distinct alterations). An additional one (3% [1/30]) distinct alteration (RICTOR) was targetable by an experimental drug in a clinical trial. As there are no FDA-approved targeted therapies for Merkel cell carcinoma and most conventional chemotherapy has been shown to be associated with poor clinical outcomes: therefore, matched targeted therapies based on molecular profiling merits investigation [91].

Our current study has some limitations. First, it was performed retrospectively with a relatively limited number of patients. Second, molecular analysis was done on archival tumor tissue, which was obtained at different time points in relationship to the clinical history; there was no information regarding the status of the patients, whether the tumors were primary or metastatic, the location of the tumor and the presence or absence of Merkel cell polyomavirus, or cytokeratin-20 positivity (found in most, but not all, Merkel cell cancers) [95]. However, despite these limitations, the genomic characterization of Merkel cell carcinomas has uncovered interesting and possibly clinically relevant results.

In summary, our 17 patients with Merkel cell carcinomas harbored 30 genomic alterations (median = 4 per patient) of which 60 were distinct molecular aberrations. The most common genomic aberrations in patients with Merkel cell carcinoma were in the *TP53* gene and the cell cycle pathway (*CDKN2A/B, CDKN2C* or *RB1*), followed by the PI3K/AKT/mTOR pathway (*AKT2, FBXW7, NF1, PIK3CA, PIK3R1, PTEN* or *RICTOR*) and DNA repair genes (*ATM, BAP1, BRCA1/2, CHEK2, FANCA* or *MLH1*). The vast majority of patients (94%) had at least one aberration that was potentially pharmacologically tractable by an FDA-approved drug or an investigational agent in a clinical trial. Indeed, of the 30 distinct genomic aberrations, 22 (73%) were potentially actionable. These observations suggest that matching patients with appropriately targeted agents is feasible and warrants study. Finally, no two patients had an identical molecular portfolio. This result is similar to that reported in metastatic breast cancer, where 131 distinct aberrations in 57 patients with no two patients having the same molecular portfolio were recently described [92–94]. Taken together, these observations suggest that customized targeted combination therapy merits investigation in patients with Merkel cell carcinoma.

## MATERIALS AND METHODS

### Patients

We investigated the genomic alterations of patients with Merkel cell carcinoma referred to Foundation Medicine (Cambridge, MA) for next-generation sequencing (December 2011 to April 2014 (*N* = 17)). Here, we report the prevalence and frequencies of these aberrations. This study was performed in accordance with University of California San Diego IRB guidelines for a de-identified database.

### Tissue samples and mutational analysis

Available tissues from diagnostic and therapeutic procedures were used to assess molecular aberrations. Samples from formalin-fixed paraffin-embedded tissue were sent for targeted next-generation sequencing at Foundation Medicine (Cambridge, MA). The test sequences the entire coding sequence of 182, or more recently 236, cancer-related genes plus 47 introns from 19 genes often rearranged or altered in cancer to an average depth-of-coverage of greater than 250X (http://foundationone.com/docs/FoundationOne_tech-info-and-overview.pdf).

This method of sequencing allows for detection of copy number alterations, gene rearrangements, and somatic mutations with 99% specificity and > 99% sensitivity for base substitutions at > five mutant allele frequency and > 95% sensitivity for copy number alterations. Foundation Medicine uses a threshold of > eight copies for gene amplification. The submitting physicians provided a diagnosis of the tumor. Next-generation sequencing data were collected and interpreted by N-of-One, Inc. (Lexington, MA; www.n-of-one.com). For the purpose of our analysis, “cell cycle pathway” aberrations included *CDKN2A/B, CDKN2C* or *RB1* alterations. Similarly, “phosphoinositide 3-kinase (PI3K)/AKT/mTOR pathway” aberrations included alterations of *AKT2, FBXW7, NF1, PIK3CA, PIK3R1, PTEN* or *RICTOR*. “DNA repair gene” abnormalities included alterations in *ATM, BAP1, BRCA1/2, CHEK2, FANCA* or *MLH1*. We have evaluated whether certain genomic alterations were actionable or not based on the availability of a drug that is approved or in clinical trials that targets that aberration with low 50% inhibitory concentration (IC_50_) or an antibody that primarily targets that abnormality.
